# Mel-18 negatively regulates stem cell-like properties through downregulation of miR-21 in gastric cancer

**DOI:** 10.18632/oncotarget.11221

**Published:** 2016-08-11

**Authors:** Xiao-Feng Wang, Xiao-Wei Zhang, Rui-Xi Hua, Yi-Qun Du, Ming-Zhu Huang, Yong Liu, Yu Fang Cheng, Wei-Jian Guo

**Affiliations:** ^1^ Department of Medical Oncology, Fudan University Shanghai Cancer Center, Shanghai, China; ^2^ Department of Oncology, Shanghai Medical College, Fudan University, Shanghai, China

**Keywords:** Mel-18, gastric cancer, cancer stem cells, miR-21

## Abstract

Mel-18, a polycomb group protein, has been reported to act as a tumor suppressor and be down-regulated in several human cancers including gastric cancer. It was also found that Mel-18 negatively regulates self-renewal of hematopoietic stem cells and breast cancer stem cells (CSCs). This study aimed to clarify its role in gastric CSCs and explore the mechanisms. We found that low-expression of Mel-18 was correlated with poor prognosis and negatively correlated with overexpression of stem cell markers Oct4, Sox2, and Gli1 in 101 gastric cancer tissues. Mel-18 was down-regulated in cultured spheroid cells, which possess CSCs, and overexpression of Mel-18 inhibits cells sphere-forming ability and tumor growth *in vivo*. Besides, Mel-18 was lower-expressed in ovary metastatic lesions compared with that in primary lesions of gastric cancer, and Mel-18 overexpression inhibited the migration ability of gastric cancer cells. Interestingly, overexpression of Mel-18 resulted in down-regulation of miR-21 in gastric cancer cells and the expression of Mel-18 was negatively correlated with the expression of miR-21 in gastric cancer tissues. Furthermore, miR-21 overexpression partially restored sphere-forming ability, migration potential and chemo-resistance in Mel-18 overexpressing gastric cancer cells. These results suggests Mel-18 negatively regulates stem cell-like properties through downregulation of miR-21 in gastric cancer cells.

## INTRODUCTION

Cancer stem cells(CSCs) refer to a small subset of cancer cells within tumors, which have the ability of self-renewal and generating diverse tumor cells [1, 2], and CSCs have a number of other biological properties that distinguish them from the remainder of tumor cells, including resistance to treatment [3], evasion of cell death [4, 5], dormancy [6] and higher metastatic ability [7], suggesting that they may play a central role in tumor recurrence and treatment failure.

Mel-18 is a member of polycomb group (PcG) proteins, which are epigenetic chromatin modifiers. Mel-18 is similar in structure but opposite in some function to another PcG member Bmi-1 [8, 9], which is a key promoter of stem cells self-renewal [10]. Mel-18 acts as a tumor suppressor and is down-regulated in some kinds of human cancers including breast cancers [11], gastric cancer [9], and prostate cancer [12]. Besides its role for differentiated cells, Mel-18 was also found to play a vital role in regulating self-renewal of stem cells [13]. Low expression of Mel-18 gave rise to the promotion of hematopoietic stem cells (HSC) self-renewal [14]. In breast cancer, Mel-18 knockdown improved the self-renewal of CSCs, and its overexpression inhibited the self-renewal activity of breast CSCs [15]. However, the roles of Mel-18 in regulating other characteristics of CSCs and in other kinds of CSCs are still unknown, and the mechanisms are unclear.

The ability of self-renewal is one key property of CSC, and a recognized experimental verification method is spheroid colony formation assay, in which cancer cells are cultured without serum, but with growth factors, such as epidermal growth factor (EGF) and recombinant basic fibroblast growth factor (bFGF) [16]. Besides self-renewal, resistance to chemotherapy and high metastasis potential are also properties of CSCs, which contribute to cancer recurrence and treatment failure. The present study aimed to investigate the functions of Mel-18 in regulating properties of CSCs from these three aspects and clarify its’ down-stream targets and mechanisms in gastric cancer.

## RESULTS

### The expression of Mel-18 correlated with stem cell markers expression and patients’ survival in gastric cancer

**Figure 1:**
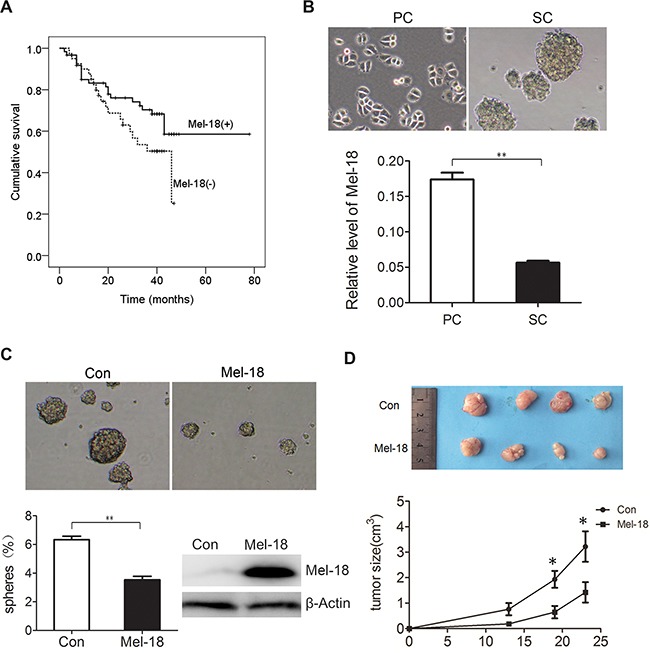
Mel-18 negatively regulated the self-renew of gastric cancer stem like cells **A.** Patients with Mel-18 positive expression survived longer than those with Mel-18 negative expression. Kaplan-Meyer survival curves were plotted as cumulative survival vs months according to Mel-18 expression (negative or positive) in cancer samples in patients with gastric cancer. The expression of Mel-18 was detected by Immunohistochemical (IHC) Assay. **B.** The expression of Mel-18 mRNA was downregulated in spheroid cells (SC) compared with that in parental adherent cells (PC). Tumorigenic spheres were derived from SGC7901 gastric cancer cell line in serum-free media containing EGF and bFGF and then photographed (upper pane). Fold change of Mel-18 in PC and SC was analyzed by QRT-PCR (lower panel). Total RNA of parental adherent cells and spheroid cells were extracted using TRIzol reagent (Invitrogen) and cDNA synthesis were performed as manufacturer's protocol. For Mel-18 mRNA, GAPDH acted as an internal control. **C.** Overexpression of Mel-18 reduced self-renew of gastric cancer stem like cells. The self-renew ability was analyzed by serum-free culture spheres formation and photographed (upper pane). The percentage of spheres formed was calculated and plotted (lower left panel), and overexpression of Mel-18 in SGC-7901 cells was determined by Western blot (*lower right panel*). Stable cell lines expressing Mel-18 was generated by transfection of Mel-18 overexpressing plasmid and selected by puromycine. Con: cells transfected with control vector; Mel-18: cells transfected with Mel-18 overexpressing plasmids. **D.** Mel-18 overexpression inhibited in vivo tumorigenecity of SGC7901 cells. In vivo tumorigenecity was detected by subcutaneously cancer cells injected SCID mice model. Suppressed tumor size after SGC7901 injected subcutaneously in one rear flank of severe combined SCID mice. Mel-18-overexpressing SGC-7901 cells or control cells were injected subcutaneously into the flanks of severe combined SCID mice. Tumor sizes were detected terminally by vernier caliper. After 4 weeks, mice were sacrificed by cervical dislocation, and tumors were removed and imaged. All experiments involving animal abided by protocols approved by the Shanghai Medical Experimental Animal Care Commission.

To explore the role of Mel-18 in gastric CSCs, we firstly detected the expressions of Mel-18 and stem cell markers or related proteins CD44, CD133, Oct4, Sox2, and Gli1 [17] in samples of 101 primary lesions of gastric cancer using immunohistochemical (IHC) assay (Supplementary Figure S1), and analyzed the correlations between Mel-18 and these stem cell markers. Results showed that expression of Mel-18 was negatively correlated with the expression of Oct4, Sox2 and Gli1 (Table 1), suggesting Mel-18 may involve in the regulation of stemness. Meanwhile Kaplan-Meier survival analysis revealed that higher expression level of Mel-18 predicted better survival (p<0.05) (Figure 1A). We also detected the expression of Mel-18, CD44, CD133, Oct4, Sox2, and Gli1 expressions in gastric cancer cells using Western blot assay or QRT-PCR (Supplementary Figure S2).

**Table 1:** The relation between Mel-18 and stem cell markers

		Oct4	Sox-2	Gli 1	CD44	CD133
Mel-18	R	−0.154	−0.283	−0.254	−0.080	−0.086
	P	0.043	0.000	0.001	0.298	0.263

R : Spearman correlation; P: P value

### Mel-18 overexpression negatively regulates stem cell-like properties in gastric cancer cells

Our former research has revealed that serum-free culture microsphere formation is available for isolating stem cell-like cells in gastric cancer. Spheroid cells overexpressed stem cell markers including Bmi-1, Oct-4, Nanog, ß-catenin, and Sox2, and acquire higher tumorigenicity, higher metastatic potential and higher chemo-resistance, suggesting micro-sphere enrich CSCs or stem cell-like cells [17]. To explore the possible role of Mel-18 in gastric CSCs, we detected the expression of Mel-18 by qRT-PCR in spheroid cells (collected after 4 weeks serum-free culture), and results revealed that lower expression of Mel-18 was found in spheroid cells than that in their parental cells(Figure 1B), suggesting the potential role of Mel-18 in suppressing the properties of CSCs. Furtherly, we established stably Mel-18 overexpressing cancer cells, and detected the sphere formation ability by serum-free culture, and found that Mel-18 overexpressing cells had lower sphere-forming ability compared with their parental cells (Figure 1C), suggesting Mel-18 inhibits self-renewal ability of gastric cancer stem like cells.

Besides sphere formation ability, higher tumorigenicity *in vivo* is also considered as self-renewal properties of CSCs, so we tested whether Mel-18 overexpression inhibited tumor growth *in vivo*. The control and Mel-18 overexpressing gastric cancer cells SGC7901 (5×10^6^total cells) were injected subcutaneously in one rear flank of severe combined immunodeficient (SCID) mice and tumor growth was examined. Mice injected with Mel-18 overexpressing cells formed smaller tumors compared to those injected with control cells within 30 days (Figure 1D). Above all, we can conclude that Mel-18 overexpression impairs the self-renewal of gastric cancer stem cell- like cells, and the similar results was also found in studies on HSC [14] and breast cancer stem cells.

**Figure 2:**
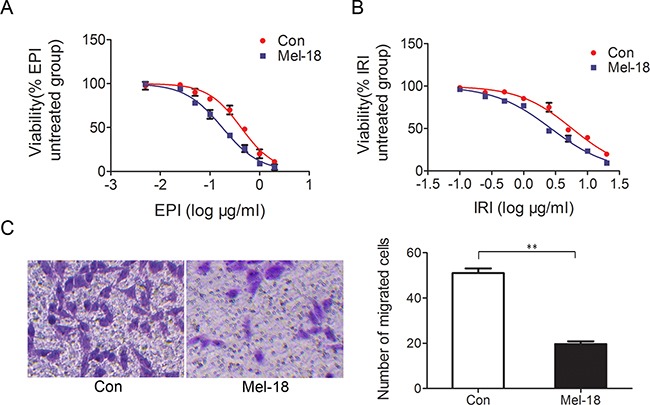
Mel-18 overexpression reduced chemotherapy resistance and metastatic potential of gastric cancer cells Growth- inhibitory curves of SGC7901 cells treated with different concentration of EPI **A.** and IRI **B.** Stable cell lines SGC7901 expressing Mel-18 were treated with different concentration of EPI and IRI, and CCK-8 assay was used to detect the number of viable cells as manufacture's procedure. The viable cell percentages were plotted as the logarithm to base 10 of the concentrate of EPI and IRI. **C.** The migrated cells number decreased in SCG7901 cells with Mel-18 overexpression (left penal: picture of migrated cells; right panel: the number of migrated cells were counted and plotted). Mel-18 overexpressing SGC7901 cells or control cells were seeded in the upper chamber of transwell without serum, while the lower compartment was added with RPMI1640 medium with 10% FBS. After 24 hours, the migrated cells were fixed with paraformaldehyde and stained with crystal violet.

Chemo-resistance is presumed to be the root of cancer treatment failure, meanwhile it is one vital property of CSCs [18]. We examined drug sensitivity by CCK-8 assay and found that Mel-18 overexpression sensitized gastric cancer cells to chemotherapy regent epirubicin (EPI) (Figure 2A) and irinotecan IRI (Figure 2B), suggesting Mel-18 negatively regulates chemo-drug resistance.

High metastasis potential is another characteristic of CSCs, and it is one primary cause of cancer death. To explore the role of Mel-18 in regulating tumor metastasis potential *in vitro*, we examined the effect of Mel-18 overexpression on gastric cancer cells migration ability by transwell migration assay. We found that Mel-18 overexpression inhibited the migration ability of gastric cancer cells (Figure 2C). Furthermore, we detected the expression of Mel-18 in primary lesions and ovary metastatic lesions of gastric cancer by IHC. In primary gastric cancer, 60.4% (61/101) samples exhibited positive staining of Mel-18, while ovary metastatic lesions expressed lower level of Mel-18 (positive rate 40.3% (29/72), P=0.009). Among those samples, 21 paired primary and metastatic lesions were included, Mel-18 was founded to be lower-expressed in ovary metastases (11/21, positive rate 52.38%), compared with that in primary lesions of gastric cancer(17/21, positive rate 80.95%, p=0.031). Taken together, the above findings revealed that Mel-18 might be a negative regulator of cancer cells migration and metastasis.

### Mel-18 downregulates miR-21, VEGF, and upregulates TIMP3 in gastric cancer cells

**Figure 3:**
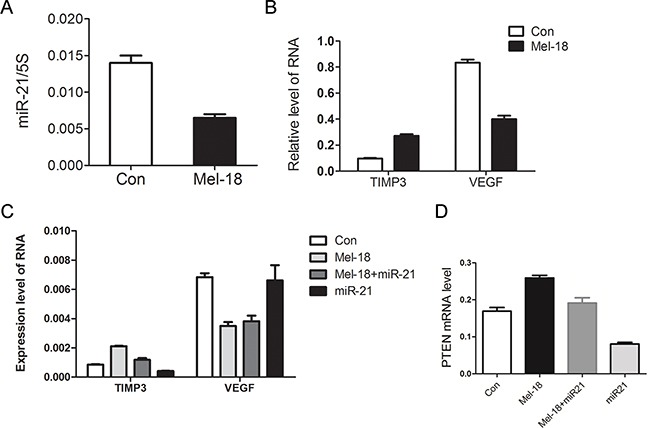
Mel-18 regulated the expression of miR-21, TIMP3, and VEGF **A.** miR-21 was downregulated by Mel-18. Fold change of miR-21 in Mel-18-overexpressing and control SGC7901 cells was analyzed by QRT-PCR. Total RNA of Mel-18-overexpressing and control SGC7901 cells was poly(A) tailed using poly(A) polymerase and then reverse-transcribed into first-strand cDNA using miRcute miRNA cDNA kit (Tiangen), and5S acted as an internal control. **B.** Overexpression of Mel-18 resulted in upregulation of TIMP3 and downregulation of VEGF. **C.** miR-21 overexpression reversed the change of TIMP expression, but not VEGF expression induced by Mel-18 overexpression. MiR-21 was overexpressed in SGC7901 cells by Lentivirus-miR-21 (Shanghai SunBio Medical Biotechnology Co., Ltd) infection. The expression of TIMP3, VEGF mRNA was analyzed by QRT-PCR in vector-infected control, Mel-18-overexpressing, co-overexpressing miR-21 with Mel-18, and miR-21-overexpressing SGC-7901 cells. **D.** miR-21 overexpression reversed the change of PTEN expression induced by Mel-18 overexpression. MiR-21 was overexpressed in SGC7901 cells by Lentivirus-miR-21 (Shanghai SunBio Medical Biotechnology Co., Ltd) infection. The expression of PTEN mRNA was analyzed by QRT-PCR in vector-infected control, Mel-18-overexpressing, co-overexpressing miR-21 with Mel-18, and miR-21-overexpressing SGC-7901 cells.

To clarify the down-stream targets and mechanisms of Mel-18 in regulating the stem cell-like properties in gastric cancer cells, we carried out a miRNAs microarray to find the potential downstream miRNAs which were regulated by Mel-18. We found that miR-21, which plays an important role in cancer development and stem like cells self-renewal [19], was downregulated by Mel-18, and this was verified by qRT-PCR (Figure 3A). We furtherly examined the relationship between the expression of Mel-18 and miR-21 in gastric cancer tissues by qRT-PCR. We found that gastric cancer lesions overexpressed miR-21 in 25/63 cases (39.7%) and lower-expressed Mel-18 in 38/63 cases (60.3%) compared to corresponding non-tumor gastric mucosal tissues, and Spearman coefficient correlation analysis showed a negative correlation between Mel-18 and miR-21 expression at RNA level (r=−0.321, P=0.009), supporting the finding of Mel-18 negatively regulating the expression of miR-21.

As VEGF and TIMP3 are important positive and negative regulator of cancer cells’ invasion and metastasis, respectively; and TIMP3 is a known target of miR-21 [20], and VEGF was reported as a downstream molecular of Mel-18 [21] and miR-21 [16], we tested whether Mel-18 regulates the expression of these two molecules, and found that Mel-18 overexpression resulted in upregulation of TIMP3 and downregulation of VEGF (Figure 3B). Then we examined whether Mel-18 regulates VEGF and TIMP3 via miR-21, and found that miR-21 downregulated TIMP3, and upregulation of TIMP3 by Mel-18 overexpression could be reversed by miR-21 overexpression (Figure 3C). However, as to VEGF, we found it was regulated by Mel-18, but not influenced by miR-21 (Figure 3C). PTEN acts as an important tumor suppressor and also play an important role in cancer stem cell self-renewal [22]. PTEN is the direct downstream target of miR-21 [23]. Then we explored whether Mel-18 regulates PTEN via miR-21, and found that miR-21 downregulated PTEN, and upregulation of PTEN by Mel-18 overexpression could be reversed by miR-21 overexpression (Figure 3D). So we concluded that Mel-18 may regulate properties of CSCs through miR-21, PTEN, TIMP3 and VEGF. Meanwhile PTEN and TIMP3 is miR-21 target, and VEGF is independent of miR-21.

### Mel-18 negatively regulates cancer stem cell-like properties through downregulation of miR-21

**Figure 4:**
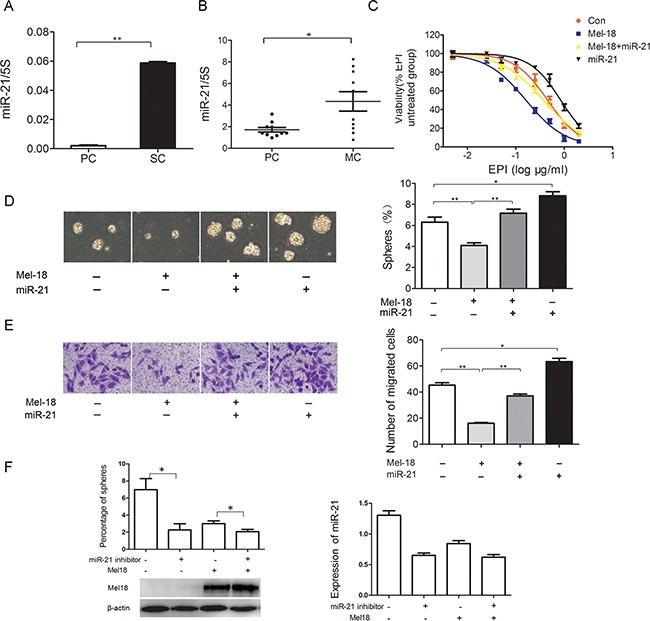
Exogenously miR-21 expression restores stem cells-like characteristics of gastric cancer cells which were inhibited by Mel-18 overexpression **A.** The elevated expression of miR-21 was found in spheroid colonies derived from SGC-7901 cells. **B.** Higher miR-21 expression was found in ovary metastatic tissues compared with its paired primary gastric cancer samples. Total RNA of primary gastric cancer samples and corresponding ovary metastatic tissues was extracted and the expression of miR-21 was analyzed as before. MiR-21 overexpression restores chemotherapy resistance **C.** self-renewal **D.** and migration potential **E.** in gastric cancer cells stablely overexpressing Mel-18. Self-renewal property, migration potential and anti-cancer drug EPI resistance were examined by spheres formation assay, Transwell migration, and CCK-8 assay, respectively. **F.** The sphere-forming ability of Mel18 in miR-21 knockdown gastric cancer cells was detected by spheres formation ability, the sphere-forming ability in co-overexpressing Mel-18 with miR-21 knockdown cells is similar to miR-21 knockdown cells.

To determine whether miR-21 play a role in regulating CSCs properties, we detected the expression of miR-21 in gastric cancer spheroid colonies from SGC-7901 and found miR-21 level was increased by 32 fold compared with its parental cells (Figure 4A). Meanwhile, we tested miR-21 expression in 10 pairs of gastric primary and metastatic cancer samples, and found miR-21 was highly expressed in ovary metastatic tissues compared with its paired primary gastric cancer samples (Figure 4B).

To further confirm the hypothesis that Mel-18 negatively regulates CSCs properties via down-regulating miR-21, we co-overexpressed miR-21 and Mel-18 in SGC-7901 gastric cancer cells. We measured sphere-forming ability, migration potential and drugs sensitivity of vector-infected control, Mel-18-overexpressing, miR-21-overexpressing, and co-overexpressing miR-21 with Mel-18 cells by using spheroid colony formation assay, Transwell chamber migration assay and CCK-8, respectively. The results indicated that miR-21 overexpression partially restored self-renewal, migration potential and chemo-resistance in gastric cancer cells overexpressing Mel-18 (Figure 4C, 4D and 4E). Here we designed a sequence targeting miR-21 and detected the sphere-forming ability of Mel18 in miR-21 knockdown gastric cancer cells, and found that the sphere-forming ability in co-overexpressing Mel-18 with miR-21 knockdown cells is similar to miR-21 knockdown cells (Figure 4F).

Collectively, these findings deduced that miR-21 participates in Mel-18-mediated regulation of CSCs’ properties.

## DISCUSSION

Gastric cancer is a heterogeneous disease and significant proportions of patients with gastric cancer are still resistant to treatment and finally die of their disease. Advances in the treatment of this disease may now come from the identification of novel therapeutic targets. more non-coding RNAs are known to paly big roles in carcinogenesis, metastasis and drug treatment resistance, including miRNA [24, 25], piRNA [26], lncRNA [27]. Results of the present study that highlighted lower expression of Mel-18 in gastric cancer tissues and stem cell-like cells, indicated that among PcG family members, Mel-18 may not only a tumor suppressor, but also an important regulator of self-renewal and other CSCs’ properties. One newest article revealed that self-renewal can be used as a therapeutic target in human colorectal cancer [10]. This study also found that miR-21 mediated the properties of CSCs as the downstream target of Mel-18, which may be a rational therapeutic target for gastric CSCs.

Mel-18 is similar in structure but opposite in function involved in carcinogenesis to another PcG member Bmi-1, a key promoter of stem cells self-renewal. In our study, we found that Mel-18 was lower-expressed in CSC-like spheroid cells and metastatic gastric cancer tissues, and Mel-18 overexpression decreased the number and size of CSC-like cells and inhibited tumorigenicity *in vivo*, decreased chemo-drug resistance and inhibited cancer cells migration. These results revealed that Mel-18 negatively regulates the CSCs properties in gastric cancer, which also opposite in function to Bmi-1 in regulating stem cells self-renewal, and the similar results was also clarified in studies on HSC and breast cancer stem cell. Kajiume [6] demonstrated that Mel-18 knockdown promoted HSC self-renewal via regulation of Hoxb4 gene expression. In breast cancer, down-regulation of Mel-18 enhanced breast CSC self-renewal ability through up-regulating Jagged-1, which was a target of WNT/TCF pathway, and a ligand and activator of Notch pathway [7]. Our research not only confirmed that Mel-18 negatively regulates the self-renewal of CSCs in a new type of human cancer, but also found that Mel-18 involved in the regulation of other CSCs properties. However, what's the mechanisms and its down-stream targets which mediated its functions? Interestingly, we screened and found miR-21 to be one of its down-stream target. Overexpression of Mel-18 resulted in down-regulation of miR-21 in gastric cancer cells and the expression of Mel-18 was negatively correlated with the expression of miR-21 in gastric cancer tissues. Furthermore, miR-21 was overexpressed in CSC-like spheroid cells and positively regulates the CSCs properties in gastric cancer cells, and miR-21 overexpression partially restored CSCs properties in Mel-18 overexpressing gastric cancer cells. These results suggest Mel-18 negatively regulates stem cell-like properties through downregulation of miR-21 in gastric cancer cells. In order to explore whether miR-21 is the main downstream target in Mel-18-mediated sphere formation. We detected the sphere-forming ability of Mel18 in miR-21 knockdown gastric cancer cells, and found that Mel-18 overexpression has no further inhibitory activity towards sphere formation, suggesting miR-21 is the main downstream target of Mel-18 in the sphere-formation process.

Furtherly, we tested whether Mel-18 regulates the expression of two miR-21 down-stream molecules, and found that Mel-18 upregulates TIMP3 via miR-21 and downregulates VEGF independent of miR-21. TIMP3 was also verified as a miR-21 target, regulated cancer cells migration, invasion, and apoptosis in different kind of cancers, such as glioma, esophageal carcinoma, renal cell carcinoma and so on [15, 18, 19].

However, as to VEGF, we found it was regulated by Mel-18, but not influenced by miR-21, which was inconsistent with previous study that miR-21 can induce VEGF expression trough PTEN/AKT signal pathway in prostate cancer cells [16]. This variance might due to the tissue diversity and Mel-18 regulating PTEN/AKT signal pathway directly and independent of miR-21 [21, 28, 29].

## MATERIALS AND METHODS

### Clinical samples

101 paraffin-embedded human gastric cancer and 72 metastatic ovary tissues including 21 paired primary and metastatic samples, and 63 frozen human gastric cancer samples and corresponding non-tumor samples were obtained from the archives of the Department of Pathology, Shanghai Cancer Center of Fudan University. Signed informed consent for the research proposes of clinical samples was obtained from every patient. The clinicopathologic variables were collected from patients’ medical records, and disease clinical stages were classified according to the 2010 UICC/AJCC gastric cancer TNM staging system.

### Cellular and molecular reagents and methods

Gastric cancer cell line SGC-7901 was cultured in RPMI-1640 supplemented with 10% fetal bovine serum (FBS) and antibiotics. Spheroid Colony Formation Assay was carried out as described previously [30]. Cells were seeded in wells (1000 cells per well or otherwise indicated) of ultra-low-attachment 6-well plates (Corning Life Sciences, Acton, MA, http://www.corning.com/lifesciences) supplemented plus2ml of DMEM/F12 medium (Invitrogen) with 10mM HEPES, human recombinant epidermal growth factor (EGF) (Invitrogen) at the concentration of 20 ng/ml, and human recombinant basic fibroblast growth factor (bFGF) (Invitrogen) at the concentration of 10 ng/ml. After 3~4 weeks, each well was examined using light microscope and spheroid colonies in 5 random fields were counted.

#### Chemo-sensitivity experiment

Cells were inoculated into 96-well plates (5000 cells per well) in triplicate supplied with RPMI-1640 medium containing 10% FBS, along with different concentrations chemotherapy reagent epirubicin (EPI) or irinotecan IRI and no drug as control. The number of viable cells was evaluated after 2 days cultivation using the Cell Counting Kit-8 (CCK8) (Dojindo, Rockville, MD, http://www.dojindo.com) following the manufacturer's instructions, and the optical absorbance at wavelength 450 nm was measured for the supernatant of each well using the plate reader Multiskan EX (Thermo Fisher Scientific Inc., Waltham, MA; http://www.thermofisher.com).

#### Cell migration assay

Cell migration ability was analyzed by the Transwell chamber assay. Cells were plated in medium without serum, and medium containing 10% FBS in the lower chamber served as chemoattractant. After 36 hours of incubation, the cells that did not migrate or invade through the pores were carefully wiped out with cotton wool. Then the inserts were stained with 20% methanol and 0.2% crystal violet, imaged, and counted with an IX71 inverted microscope (Olympus).

### Virus production and infection

Stable cell lines expressing Mel-18 was generated by transfection of Mel-18 overexpressing plasmid and selected by puromycine as described previously [31]. Lentivirus-miR-21 was generated by Shanghai SunBio Medical Biotechnology Co., Ltd and the infection was conducted as manufacturer's protocol.

### Quantitative real time RT-PCR (QRT-PCR) assays

Total RNA of cultured cell lines and tissue samples were extracted using TRIzol reagent (Invitrogen). For Mel-18, CD133, Oct4, Gli1, Sox2, TIMP3, VEGF and PTEN mRNA, GAPDH acted as an internal control. As to miR-21, total RNA was poly(A) tailed using poly(A) polymerase and then reverse-transcribed into first-strand cDNA using miRcute miRNA cDNA kit (Tiangen).5S acted as an internal control and the SYBR Green-based real-time PCR was conducted using 7900HT fast real-time PCR System (Applied Biosystems).

The primer of related gene for qRT-PCR as follows:

**Table:** 

Gene	Forward primer	Reverse primer
Mel-18	5′-GATGGATGTG CCCAGCAAGT-3′	5′-GGAGCCTTGT CGCTGACTGA-3′
CD133	5′-TTACGGCA CTCTTCACCT-3′	5′-TATTCCACA AGCAGCAAA-3′
Oct4	5′-GTGGAGAGC AACTCCGATG-3′	5′-CTCACTCGGTTCT CGATACTGGTTC-3′
Gli1	5′-TTCCTACCAG AGTCCCAAGT-3′	5′-CCAGCCCCAGC GTCAAAGGTG-3′
Sox2	5′-CGAGATAAACA TGGCAATCAAT-3′	5′-ATTCAGCAAGA AGCCTCTCCTT-3′
GAPDH	5′-GCTGAACGG GAAGCTCACTG-3′	5′-GTGCTCAGTG TAGCCCAGGA-3′
PTEN	5′-TTGAAGACCATA ACCCACCACAG-3′	5′-GGCAGACCACA AACTGAGGATTG-3′
TIMP3	5′-CAGGTCGCGT CTATGATGGCC-3′	5′-AGGTGATACC GATAGTTCAGCC-3′
VEGF	5′-GGAGTACCCTGA TGAGATCGAGT-3′	5′-GTCACATCTG CAATGACGTTCG-3′

### Transplantation of cancer cells in vivo

SGC-7901 cells transfected with either Mel-18-overexpressing plasmid or the mock plasmid were injected subcutaneously into the flanks of SCID mice. Tumor sizes were detected terminally by vernier caliper. After 4 weeks, mice were sacrificed by cervical dislocation, and tumors were removed and imaged. All experiments involving animal abided by protocols approved by the Shanghai Medical Experimental Animal Care Commission.

### Immunologic reagents and methods

Mel-18 was detected in cell lysate and tumor tissues with western blot (WB) and immunohisto-chemistry (IHC), respectively, as standard procedures [32]. For incubation, the primary antibody against Mel-18 was diluted with ratio 1:1000 for WB and 1:100 for IHC. Immunohistochemical (IHC) analyses was used to detect the expression of stem cell markers Mel-18, Oct4, Sox2, Gli1, CD44, CD133 in samples of GC primary lesions. IHC was performed by using a highly sensitive streptavidin-biotin-peroxidase detection system. All slides were interpreted by two independent observers in a blinded fashion. More than 10% of the cells stained with moderate or strong staining intensity were considered positive. Otherwise, the sample was considered negative.

### Statistical analysis

All data were shown as mean ± SEM, the Student t test was used for statistical analysis unless otherwise noted, with P < 0.05 considered significant. In IHC assays of GC samples, Spearman's Rank correlation assay was used to determine the correlation between Mel-18 and stem cell markers expression. In QRT-PCR analysis of fresh tissues, the correlation between Mel-18 and miR-21 expression levels was analyzed by the Pearson coefficient test.

## SUPPLEMENTARY FIGURES


