# Supplementary material for: Mel-18 negatively regulates stem cell-like properties through downregulation of miR-21 in gastric cancer
Source: Oncotarget. 2016 Aug 11;7(39):63352–61. doi: 10.18632/oncotarget.11221 (PMC5325369; doi:10.18632/oncotarget.11221)
Supplement: Supplementary file 1 [file oncotarget-07-63352-s001.pdf]

# Mel-18 negatively regulates stem cell-like properties through downregulation of miR-21 in gastric cancer

## SUPPLEMENTARY FIGURES

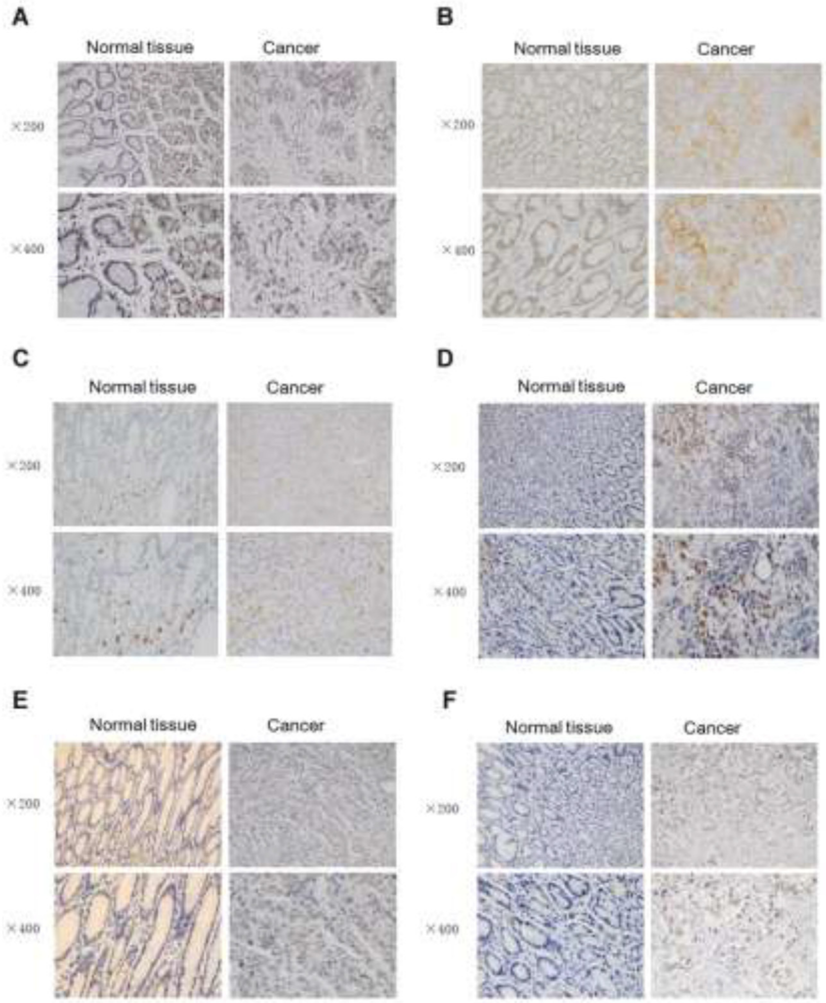


**Supplementary Figure S1: Representative figures of Mel-18 and several CSC-related proteins in gastric tumors and its surrounding normal tissues.** **A.** cancer tissues express less Mel-18 compared with the normal tissues. In panel **B.** cancer tissues express more CD44 compared with normal tissues. **C.** cancer tissues expresses more CD133 compared with normal tissues. **D.** cancer tissues expresses more Oct4 compared with normal tissues. **E.** cancer tissues expresses more SOX2 compared with normal tissues. **F.** cancer tissues expresses more Gli1 compared with normal tissues.

A

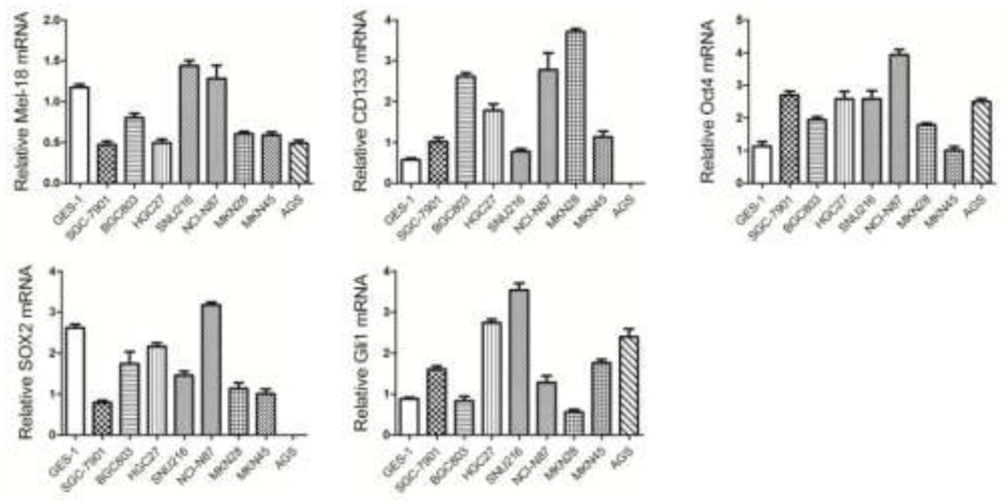


B

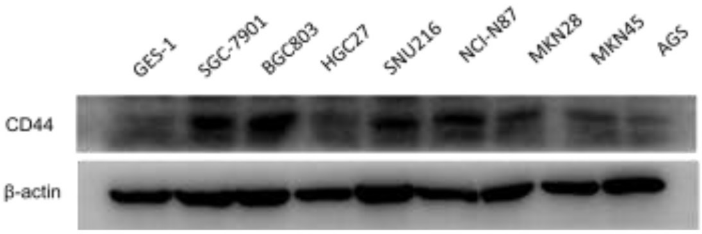


**Supplementary Figure S2: The expression of Mel-18, CD44, CD133, Oct4, Sox2 and Gli1 using Western blot or qRT-PCR method in seven gastric cancer cell lines. A.** The expression of Mel-18, CD133, Oct4, Sox2 and Gli1 using qRT-PCR method in seven gastric cancer lines. **B.** The expression of CD44 using Western blot method.
